# Antimicrobial peptidase lysostaphin at subinhibitory concentrations modulates staphylococcal adherence, biofilm formation, and toxin production

**DOI:** 10.1186/s12866-023-03052-z

**Published:** 2023-10-26

**Authors:** Yuan Yue, Ke Chen, Changfeng Sun, Sarfraz Ahmed, Suvash Chandra Ojha

**Affiliations:** 1 Department of Infectious Diseases, The Affiliated Hospital of Southwest Medical University, Luzhou, 646000 Sichuan China; 2 National Engineering Laboratory for Resource Development of Endangered Crude Drugs in Northwest China, Shaanxi Normal University, Xi’an, China; 3 Southwest Medical University, Jiangyang District, Luzhou, 646000 Sichuan China; 4 Wellman Centre for Photomedicine, Massachusetts General Hospital, Harvard Medical School, Boston MA 02114, USA

**Keywords:** APLss, Staphylolytic activity, Cell adhesion, Biofilm inhibition, Transcriptional change

## Abstract

**Background:**

The ability of antimicrobial agents to affect microbial adherence to eukaryotic cell surfaces is a promising antivirulence strategy for combating the global threat of antimicrobial resistance. Inadequate use of antimicrobials has led to widespread instances of suboptimal antibiotic concentrations around infection sites. Therefore, we aimed to examine the varying effect of an antimicrobial peptidase lysostaphin (APLss) on staphylococcal adherence to host cells, biofilm biomass formation, and toxin production as a probable method for mitigating staphylococcal virulence.

**Results:**

Initially, soluble expression in *E. coli* and subsequent purification by immobilized-Ni^2+^ affinity chromatography (IMAC) enabled us to successfully produce a large quantity of highly pure ~ 28-kDa His-tagged mature APLss. The purified protein exhibited potent inhibitory effects against both methicillin-sensitive and methicillin-resistant staphylococcal strains, with minimal inhibitory concentrations (MICs) ranging from 1 to 2 µg/mL, and ultrastructural analysis revealed that APLss-induced concentration-specific changes in the morphological architecture of staphylococcal surface membranes. Furthermore, spectrophotometric and fluorescence microscopy revealed that incubating staphylococcal strains with sub-MIC and MIC of APLss significantly inhibited staphylococcal adherence to human vaginal epithelial cells and biofilm biomass formation. Ultimately, transcriptional investigations revealed that APLss inhibited the expression of *agrA* (quorum sensing effector) and other virulence genes related to toxin synthesis.

**Conclusions:**

Overall, APLss dose-dependently inhibited adhesion to host cell surfaces and staphylococcal-associated virulence factors, warranting further investigation as a potential anti-staphylococcal agent with an antiadhesive mechanism of action using in vivo models of staphylococcal toxic shock syndrome.

**Supplementary Information:**

The online version contains supplementary material available at 10.1186/s12866-023-03052-z.

## Introduction

Microbial adhesion to epithelial cell surfaces can be considered one of the first steps in colonization and invasive disease. *Staphylococcus aureus*, notably Methicillin-resistant *S. aureus* (MRSA) is an opportunistic pathogen that frequently colonizes human skin and mucosal membranes, including the vagina, with vaginal colonization reaching almost 25% in childbearing populations [1, 2]. Staphylococcal vaginal colonization can lead to vaginitis, and during pregnancy, bacterial ascension into the upper reproductive tract can lead to adverse birth outcomes. Staphylococcal strains also produce a wide range of toxins, including enterotoxins, toxic shock syndrome toxins, hemolysin, leukocidin, and exfoliative toxins, which cause diseases ranging from minor skin infections to systemic life-threatening conditions such as toxic shock syndrome (TSS) [3]. Considerably over the past decade, there has been a growing realization that staphylococcal biofilms are a cause of great concern in a variety of infections [4]. In order to form biofilms, staphylococcal cells produce a polymer-based extracellular matrix made up of proteins, carbohydrates, and extracellular DNA, which shields them in a sticky matrix and allows them to survive in hostile environments [5]. The formation of biofilms reduces bacterial susceptibility to antimicrobial agents and immune defenses, making eradication more difficult.

In the treatment of infections, caused by staphylococcal strains, several antibiotics are being used including penicillin, methicillin, vancomycin, and linezolid depending on the strain responsible for the infection [6, 7]. Vancomycin remains the cornerstone of empirical treatment for systemic MRSA infections. Nevertheless, prolonged hospitalization, recurrent infection following therapy, high rates of clinical failures, nephrotoxicity, and a high incidence of non-susceptible strains, constrain its efficacy [8–11]. Besides that, numerous non-traditional initiatives for treating and preventing MRSA infections are being investigated, including antimicrobial peptides, [12] natural products, [13] and anti-staphylococcal vaccines; [14] however, there are significant challenges in developing these agents and bringing them from bench-to-bedside. Of note, while reports of vancomycin failure have emerged for vancomycin intermediate-resistant *S. aureus* (VISA) or heterogeneous VISA (hVISA), no data indicate improved outcomes with existing antimicrobial agents; therefore, alternative antimicrobial agents for staphylococcal infections are of utmost importance.

**Fig. 1:**
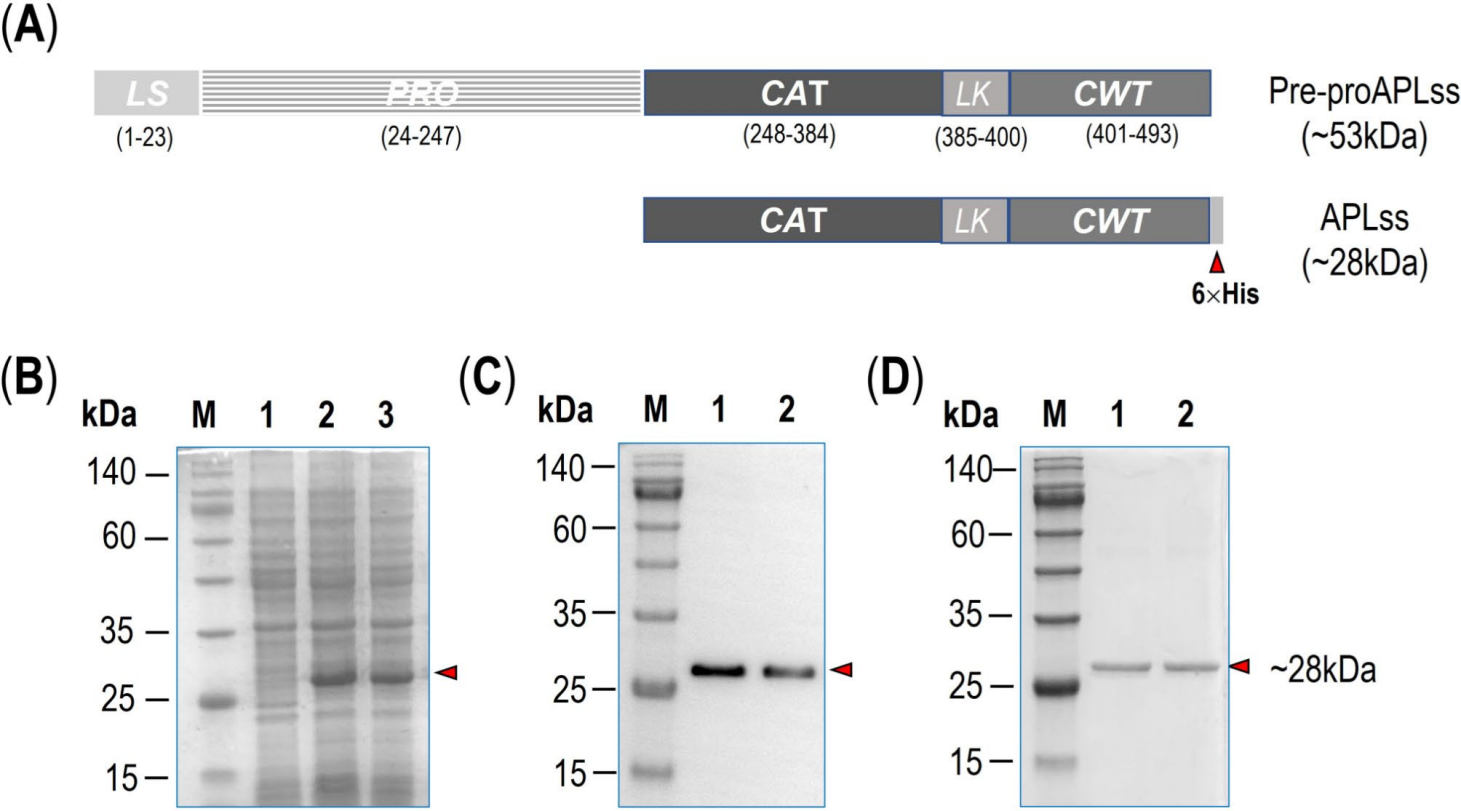
**(A)** Schematic representation of the full length pre-proAPLss (∼53-kDa) displaying the leader sequence (LS), proregion (PRO), catalytic domain (CAT), linker (LK), and cell wall-targeting domain (CWT). The ∼28-kDa APLss was incorporated with a 6× His tag at the C terminus. **(B)** SDS-PAGE (Coomassie brilliant blue-stained 12% gel) analysis of crude extracts from *E. coli* cells expressing APLss (lane 2), uninduced *E. coli* cells as a negative control (lane 1), the soluble fraction of cell lysate after centrifugation (lane 3), and M represents protein marker. **(C)** The corresponding Western blot probed with anti-His tag antibody followed by ALP-conjugated secondary antibody. M, protein marker; Lane 1, *E. coli* lysate fraction containing APLss; and Lane 2, *E. coli* soluble fraction containing APLss. **(D)** SDS-PAGE (12% gel) of the purified APLss protein via IMAC (lane 1 and 2) and M represents protein marker

Antimicrobial peptidase lysostaphin (APLss), a class III bacteriocin, is a Zn^2+^-dependent antibacterial endopeptidase produced by *Staphylococcus simulans* subsp. staphylolyticus that cleaves pentaglycine cross-bridges in certain staphylococci cell walls [15]. The catalytic Zn^2+^ plays an important role in cleaving inter-peptide bridges in the staphylococci cell wall by activating a water molecule to act as a nucleophile [16]. The enzyme is produced as a 493-amino-acid pre-proenzyme that includes a leader sequence (residues 1–23), a tandem-repeat region (residues 24–27), a Zn^2+^-comprising catalytic domain (residues 246–384), a flexible linker (residues 385–400), and a cell wall-targeting (CWT) domain (residues 401–493) (*see* Fig. 1A) [17]. During in vivo maturation, the signal sequence and tandem repeats are eliminated, resulting in an active peptidase lysostaphin of ~ 28 kDa [18]. APLss is very effective against both actively growing and dormant cells of staphylococci, making it superior to chemical antibiotics, which may either kill or inhibit the growth of bacteria [19]. In other studies, APLss has been shown to have a synergistic effect with LysK, the staphylococcal bacteriophage K endolysin, in killing MRSA strains, [20] suggesting that it could be combined with antibiotics or other peptidolytic enzymes to improve the therapeutic potential in treating both multidrug-resistant and chronic staphylococcal infections.

The effect of antimicrobial agents has been established in terms of concentration levels that limit or kill pathogens in vitro or under experimental settings. Antimicrobial concentrations lower than the MIC (sub-MIC) may induce alterations in bacterial features in vitro and in vivo, including physiological and biochemical functions [21, 22]. It has previously been demonstrated that subinhibitory doses of certain antibiotics modulate the expression of virulence determinants by *S. aureus*, which may alter disease severity [23, 24]. In this regard, Jin et al [25] reported that mupirocin at sub-MIC levels causes biofilm formation in *S. aureus*. Besides that, another study revealed that clindamycin, which is used to treat Gram-positive bacterial infections, induces *S. aureus* biofilm development at sub-MIC levels [26]. Additionally, antibiotics with sub-MICs alter bacterial interactions with host cell surfaces, as evidenced by lower host cell adhesion [22]. Based on multiple series of in vitro evidence, guidelines issued recently advocate using antibiotics that suppress virulence factor expression for the management of severe illnesses [27]. Therefore, staphylococcal infection management has been complicated not only by rising resistance to current antibiotics but also because of the necessity to influence bacterial virulence to attain therapeutic success.

In this present study, we achieved a simple approach for producing a large amount with high purity of the ~ 28-kDa His-tagged APLss via IMAC purification of the soluble tagged protein highly expressed in *E. coli*. The purified APLss was then tested for staphylolytic activity against methicillin-sensitive *S. aureus* (MSSA) and methicillin-resistant staphylococcal strains, as well as its impact on ultrastructural integrity. Since subinhibitory concentrations of antibiotics affect the virulence properties of microorganisms in various ways, we also studied the staphylococcal adhesion to the normal human vaginal epithelial cells (HVEC), biofilm formation, and transcriptional changes in presence of subinhibitory and inhibitory doses of APLss. The findings gathered here provide experimental evidence for the subinhibitory effect of APLss on modulating virulence factors for potential therapies, paving the way for further study as a potential anti-staphylococcal agent with an antiadhesive mechanism of action using in vivo models of staphylococcal TSS.

## Materials and methods

### Bacterial strains and growth conditions

Four staphylococcal ATCC reference strains, including MSSA 25923, MSSA 29213, MRSA 43300, and MRSA 33591, were employed in this study. The reference strains were stored at − 80 °C in Luria Bertani (LB) Broth (Solarbio Technology Co. Ltd., Beijing, China), supplemented with 25% (v/v) glycerol (G-CLONE, Beijing, China). All bacterial strains were grown on LB medium and incubated overnight at 37 °C under aerobic conditions before the assays.

### Cell culture

The HVEC cell line was purchased from Procell Life Science and Technology Co., Ltd., Wuhan, China. The cells were grown in DMEM medium (Servicebio Technology Co., Ltd, Wuhan, China) supplemented with 10% (v/v) Fetal Bovine Serum (FBS) (G-CLONE, Beijing, China) and maintained in a humidified incubator at 37 °C with 5% CO_2_ to attain confluence. The cells were passaged in 75 cm^2^ flasks (Servicebio Technology Co., Ltd, Wuhan, China), counted using a hemocytometer, and seeded into 12-well tissue culture plates (Servicebio Technology Co. Ltd, Wuhan, China) for subsequent procedures.

### Construction of the recombinant plasmid with his-tagged fusion

A 740-bp gene (APLss) encoding the ~ 28-kDa mature APLss of *S. simulans* with an added 6×His sequence at the c-terminal was custom-synthesized in pET17d expression vector with *NcoI* and *BamHI* restriction sites, yielding pLss-246 M/H6 plasmid (DongXuan Gene Technology Co. Ltd., Jiangsu, China). The resulting plasmid was transformed into *E. coli* strain JM109 for plasmid verification by restriction endonuclease digestion and DNA sequencing before being retransformed into an expression host *E. coli* strain BL21 (DE3).

### Protein expression and characterization

BL21 (DE3) *E. coli* cells containing pLss-246 M/H_6_ were grown at 37 °C in LB broth medium supplemented with 100 µg/mL ampicillin until OD_600_ of the culture reached ∼0.6, and then protein expression was induced with 0.1 mM isopropyl-β-D-thiogalactopyranoside and incubated further for another 4 h. Cells expressing APLss were collected via centrifugation (6000 × g, 4 °C, 15 min) and resuspended in 20 mM HEPES [4-(2-hydroxyethyl)-1-piperazineethanesulfonic acid] buffer (pH 7.4) comprising 1 mM phenylmethylsulfonyl fluoride. The induced cells were then ultrasonically disrupted using a VCX 750-Sonics Vibra CellTM (Sonics & Materials, Inc., Newtown, USA) with the following settings: 5 cycles of amplitude 30%, 5-s ON, 3-s OFF, and a total time ON (1 min/cycle). The total lysate and supernatant were separated by centrifugation (12,000 × *g*, 4 °C, 20 min) and examined by sodium dodecyl sulfate-polyacrylamide gel electrophoresis (SDS-PAGE, 12% w/v).

### Western blotting analysis

Following SDS-PAGE, proteins separated by molecular weight were electroblotted onto a polyvinylidene difluoride (PVDF) membrane. The membrane was serially probed with mouse anti-His tag monoclonal antibody (1:3000 dilution, Invitrogen, Waltham, MA, USA) and alkaline phosphatase (ALP)-conjugated goat anti-mouse antibody (1:5000 dilution, Invitrogen, Waltham, MA, USA) following blocking with 5% skim milk in phosphate-buffered saline (PBS, 120 mM NaCl, 16 mM Na_2_HPO_4_, 4 mM NaH_2_PO_4_, pH 7.4). Finally, color was developed using 5-bromo-4-chloro-3-indolyl phosphate/nitro blue tetrazolium substrates (G-CLONE, Beijing, China).

### Protein purification assay

Upon ultrasonically disrupted induced cells centrifugation, the supernatant of the total lysate was loaded onto an affinity-based Ni^2+^-NTA column (5-mL HisTrap™ HP, GE Healthcare Bio-Sciences AB, Uppsala, Sweden) pre-equilibrated with 20 mM HEPES buffer (pH 7.4) comprising 200 mM NaCl and 10 mM imidazole (IMZ). Unbound proteins were removed with 20 mM HEPES supplemented with 30, 50, 70, and 100 mM IMZ at a flow rate of 1 mL/min in at least 5 column volumes for each concentration. His-tagged proteins were eluted stepwise with 150 and 250 mM IMZ at a flow rate of 1 mL/min, respectively. Fractions containing the His-tagged APLss were pooled after SDS-PAGE analysis, concentrated by ultrafiltration (10-kDa cut-off, Solarbio Technology Co. Ltd., Beijing, China), and desalted through a Zeba Spin desalting column (7-kDa cut-off, ThermoFisher Scientific, Shanghai, China). Purified APLss protein concentrations were measured using Bradford microassay in 20 mM HEPES buffer (pH 7.4) (ThermoFisher Scientific, Shanghai, China).

### Antimicrobial assay

MIC assays were performed in 96-well microtiter plates, as previously described [28]. Briefly, target strains were cultivated overnight under the optimal conditions and medium, subcultured into the fresh broth, and allowed to grow to an OD_600_ of ∼0.5. In a 96-well plate containing 2-fold serially diluted antimicrobial agents, the cell suspension was diluted to a final inoculum of roughly 2 × 10^5^ CFU/ml in a volume of 0.2 ml. Following a 16-hour incubation at 37 °C, the MIC was determined as the lowest bacteriocin concentration that inhibited visible growth. Cell suspensions from the MIC test were plated onto LB agar to determine the minimal bactericidal concentration (MBC). The concentration of bactericidal agent at which no bacterial colony could be seen on an LB agar plate after 24 h of incubation at 37 °C in aerobic conditions was regarded as the MBC.

### Time-kill assay

The anti-staphylococcal activity of APLss was verified using a turbidity reduction assay that was largely based on the methods previously described [29]. In brief, a single colony from an overnight culture was resuspended in 5 mL LB broth and aerobically incubated at 37 °C ^◦^ for 12 h. Following overnight incubation, 100 µL of aliquots were transferred to a new LB broth and incubated for an additional 4 h to dilute the pre-formed toxin effect. The purified APLss was diluted to desired concentrations with HEPES buffer before being incubated with 100 µL of *S. aureus* cultures (initial OD_600_ of 0.4, ∼10^6^]. CFU/mL) in a flat-bottomed 96-well microtiter plate. The OD_600_ of the tested cell suspension was monitored every 20 min during the 2-hour incubation period. Cell cultures treated with 20 mM HEPES buffer served as a negative control (pH 7.4).

### Scanning electron microscopy (SEM)

Reference strains of staphylococci, including MSSA 25923 and MRSA 33591 were examined by SEM to determine the effect of APLss on cell morphology and ultrastructure as described previously [30]. Briefly, staphylococcal suspensions were incubated with 0.5× and 1× MIC of APLss for 12 h at 37 °C before being collected and resuspended in PBS (pH 7.4). Bacterial cells were first fixed with a mixture of 2.5% glutaraldehyde and 4% paraformaldehyde for 4 h, and then with a cross-linking reagent, 1% osmium tetraoxide, for 1 h. The specimens were then dehydrated with varying ethanol concentrations, critical point dried with carbon dioxide, stacked on a stub, and sputter-coated with gold. The specimens were examined with a JEOL JSM 6400 SEM (JEOL Ltd., Tokyo, Japan).

### Adhesion assay

The adhesion test was performed using the previously described method [31]. Briefly, subconfluent HVEC cell (Procell Life Science and Technology Co., Ltd., Wuhan, China) monolayers were infected with a 1:200 cell-to-bacterial ratio suspension in a 12-well cell culture plate and incubated at 37 °C for 1 h. Following incubation, cells were washed three times with PBS at pH 7.4 (Solarbio Technology Co. Ltd., Beijing, China). To facilitate dissolution and as a negative control in adhesion experiments, PBS was used. The cytotoxic effect of the tested antistaphylococcal agents against the HVEC was determined using the crystal violet staining [31]. To lyse the eukaryotic cells and detach the adherent bacteria, 100 µL of 1% Triton X-100 was added and incubated at room temperature for 10 min before adding 900 µL of LB broth medium. Subsequently, the number of bacteria adhering to the HVEC cells was determined by plating serially diluted suspensions on LB-agar plates and counting the colonies.

### Biofilm formation assay

Biofilm formation was determined using the previously described method [32]. Briefly, staphylococcal strains were diluted 1000-fold into fresh LB broth medium supplemented with 0.1 M glucose and then incubated in a flat-bottomed 96-well plate with defined concentrations of APLss at 37 °C for 24 h. The negative control was staphylococcal cell suspensions treated without APLss, and the positive control was the vancomycin inhibitory concentration (1× MIC). Following 24 h of incubation, the planktonic cells were carefully removed from the 96-well plate and gently washed with PBS. The biofilms were stained for 30 min at room temperature with 0.1% crystal violet (CV) (Solarbio Technology Co. Ltd., Beijing, China), and the wells were rinsed twice with PBS to remove any residual staining dye. The stained biofilms were treated with 100 µL of a 1:1 ethanol and acetone solution, and the absorbance of each sample was measured using a microplate reader at 565 nm.

### Confocal laser scanning microscopy (CLSM) observation of biofilm formation

The staphylococcal reference strains, including MSSA 25923 and MRSA 33591 were examined by CLSM to determine the effect of APLss on biofilm formation as described previously [33]. The overnight bacterial cultures were diluted 1000× with LB broth and exposed to 0.25×, 0.5×, and 1× MIC of APLss before incubating for 24 h in a CLSM-specific culture dish (NEST Biotechnology Co., Ltd., China). The negative control was staphylococcal cell suspensions without APLss, while the positive control was vancomycin 1× MIC. Following incubation, non-adherent bacterial cells were gently removed and washed twice with PBS to remove any residual floating cells. The dish was then covered with 1 mL of 2.5% glutaraldehyde solution, and the biofilm cells were fixed for 4 h. After gently removing the fixative solution and two PBS washes, 1 mL (10 µg/mL) fluorescein isothiocyanate (FITC; Sigma, USA) was added to the dish, and biofilms were stained for 30 min at 4 °C in the dark. The solution was removed, washed twice with PBS, and the cells were stained for 15 min at 4 °C in the dark with propidium iodide (PI). Finally, the biofilm cells were observed using CLSM after two more washes with PBS.

### Biofilm degradation assay

Biofilm degradation was determined using the previously described method with slight modification [32]. Briefly, staphylococcal strains diluted into a new LB medium supplemented with 0.1 M glucose were incubated in a flat-bottomed 96-well plate. After 24 h aerobic incubation at 37 °C, the planktonic cells were removed, and the established biofilm that adhered to the flat-bottomed 96-well plate was thoroughly washed with PBS before being treated with 0.25×, 0.5×, and 1× MIC of APLss. The untreated established biofilm served as the negative control, while vancomycin inhibitory concentration (1× MIC) served as the positive control. Following 16 h of incubation, cell suspensions were carefully removed from the 96-well flat-bottomed plate, and the established biofilms were gently rinsed with PBS and stained with 0.1% crystal violet for 30 min at room temperature (Solarbio Technology Co. Ltd., Beijing, China). The wells were rinsed twice with sterile water to get rid of any residual staining dye. The stained biofilms were exposed to 100 µL of a 1:1 ethanol and acetone solution, and the absorbance was measured using a microplate reader at 565 nm. The relative change in OD_600_ of established biofilm for different APLss concentrations compared to the untreated control was extrapolated by asserting that the untreated biofilm was 100%.

### Virulence gene expression analysis

Transcriptional changes of virulence genes were determined using the previously described method with slight modification [6]. Staphylococcal strains were cultivated in LB medium overnight, then subcultured into the fresh broth, and allowed to expand to an OD_600_ of ~ 0.5. With 0.5× and 1× MIC of APLss, the cell culture was diluted to a final inoculum of approximately 2 × 10^5^ CFU/ml in a volume of 1.5 ml. The negative control was a cell suspension without APLss. Following a 6-hour incubation at 37 °C, total RNA was extracted from the cells using the TRIzol reagent (G-CLONE, Beijing, China). The recovered RNA was then treated with RNase-free DNase for 10 min. A 260/280 nm absorbance ratio was determined using a NanoDrop spectrophotometer (Thermo Scientific, Waltham, MA) to quantify the amount and purity of total RNA. To reverse transcribe total RNA into cDNA, a First Strand cDNA Synthesis Kit was employed. The obtained cDNA was utilized as a template for real-time amplification (LightCycler 2.0; Roche) with SYBR Green Real-Time PCR Master Mix (Servicebio Technology Co., Wuhan, China) and the specific primers listed in Table 1. The reaction program consisted of 95 °C for 30 s, 40 cycles of 95 °C for 15 s, 55 °C for 10 s, 72 °C for 30 s, and a final extension of 72 °C for 5 min. The housekeeping gene 16sRNA gene served as a reference gene and the relative expression of virulence genes was calculated using the 2^−△△Ct^ method.

**Table 1:** List of genes and primer sequences used in the present study

Genes	Sense primers (5′–3′)	Antisense primers (5′–3′)
*sea*	ATGGTGCTTATTATGGTTATC	CGTTTCCAAAGGTACTGTATT
*agrA*	TGATAATCCTTATGAGGTGCTT	CACTGTGACTCGTAACGAAAA
*hla*	TCCAGTGCAATTGGTAGTCA	GGCTCTATGAAAGCAGCAGA
*spa*	TATGCCTAACTTAAATGCTG	TTGGAGCTTGAGAGTCATTA
*16S rRNA*	GCTGCCCTTTGTATTGTC	AGATGTTGGGTTAAGTCCC

### Statistical analysis

Unless otherwise specified, all statistical analyses were conducted on data from at least three separate experiments. Comparisons between experimental conditions and their respective controls were analyzed statistically using one-way analysis of variance (ANOVA) in GraphPad Prism 8.3.1 (GraphPad Software Inc., La Jolla, CA, USA). P-values < 0.05 imply a statistically significant difference.

## Results

### Expression, verification, and purification of the ~ 28-kDa his-tagged APLss protein

The ~ 28-kDa His-tagged target protein was overexpressed in *E. coli* BL21(DE3) from a plasmid clone (pAPLss-246 M/H6) containing the APLss-encoding gene segment under the control of the T7 promoter. SDS-PAGE analysis of *E. coli* lysate prepared in protein buffer revealed that the resulting protein had a mass of ~ 28 kDa (Fig. 1B, lane 2) and was vastly produced in *E. coli* cells as a soluble form (Fig. 1B, lane 3). The expressed protein was verified via Western blotting by probing the blotted proteins on the PVDF membrane with the anti-6× His antibodies, confirming the presence of 6× His-tag at their C-terminal end (Fig. 1C, lanes 1 and 2). Subsequently, the recombinant 6× His-tagged proteins in *E. coli* lysate supernatant were purified by single-step affinity-based chromatography using Ni^2+^-NTA HisTrap column. Stepwise elution of His-tagged target proteins was performed at a flow rate of 1 mL/min using 150 mM and 250 mM IMZ, respectively. SDS-PAGE analysis of the eluted fractions revealed that ∼12 mg of the 28-kDa His-tagged target APLss with > 98% purity was obtained from the crude preparation of 1-L bacterial cell culture (Fig. 1D, lanes 1 and 2). Taken together, it is obvious that our present strategy for soluble expression and IMAC purification of the His-tagged mature APLss is fairly effective in terms of recovery yield, purity, and ease.

### APLss dose-dependently induces staphylolytic activity

**Fig. 2:**
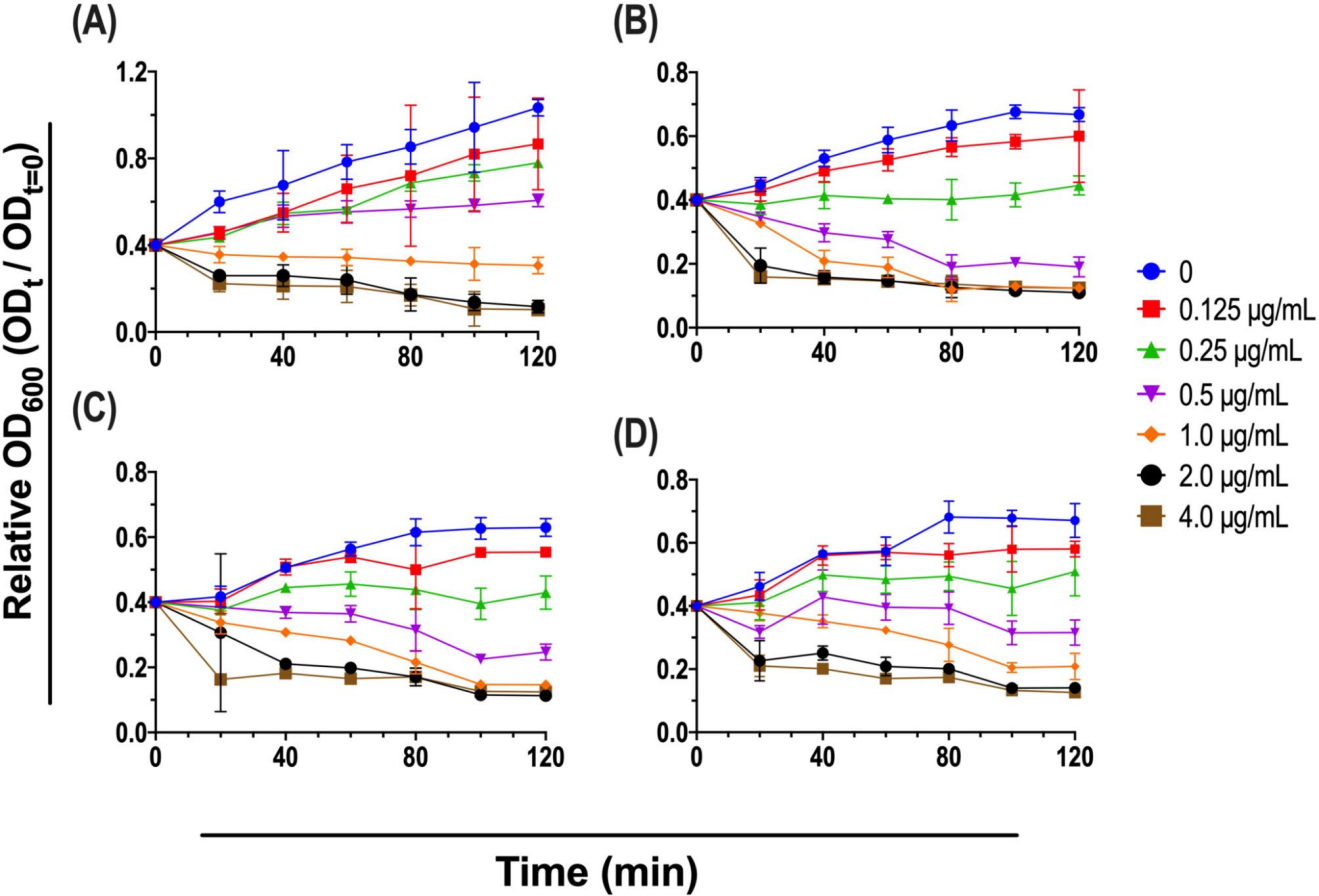
Staphylolytic activity of purified APLss at various concentrations as determined by a turbidity reduction assay against reference strains including **(A)** MSSA 25923, **(B)** MSSA 29213, **(C)** MRSA 33591, and (4) MRSA 43300. Abbreviations: min, minutes; OD, optical density; t, final time; t = 0, initial time

The antimicrobial properties of the APLss on four reference staphylococcal strains were investigated by measuring MIC values. Table 2 summarizes the MICs of APLss and the recommended empirical antibiotic vancomycin for staphylococcal infection. Among all strains examined, APLss had the lowest MICs. The MICs for lysostaphin ranged from 1.0 to 2.0 µg/ml, whereas those for vancomycin were from 2.0 to 4.0 µg/ml. MBC testing was concurrently performed to demonstrate the bactericidal effect of APLss and vancomycin, which showed that the MBC for both antimicrobials was at least twice the respective MICs (Table 2). The efficacy of APLss on staphylococcal growth was further studied using in vitro time-kill assays in which relative growth was assessed by measuring absorbance (OD_600_) at time t and compared to time t = 0 and was plotted against time. A noticeable decrease in bacterial viability was observed as shown by the steep reduction in the slope (Fig. 2). In accordance with the MIC findings, exposure to APLss at 1× MIC doses effectively inhibited the growth of all four reference strains.

**Table 2:** Antimicrobial activity of APLss and vancomycin against reference strains

	APLss			Vancomycin	
ATCC strains	MIC (µg/mL)	MBC (µg/mL)		MIC (µg/mL)	MBC (µg/mL)
MSSA 25923	2	4		4	8
MSSA 29213	1–2	4		4	8
MRSA 43300	1–2	4		2–4	8
MRSA 33591	2	4		4	4–8

**Fig. 3:**
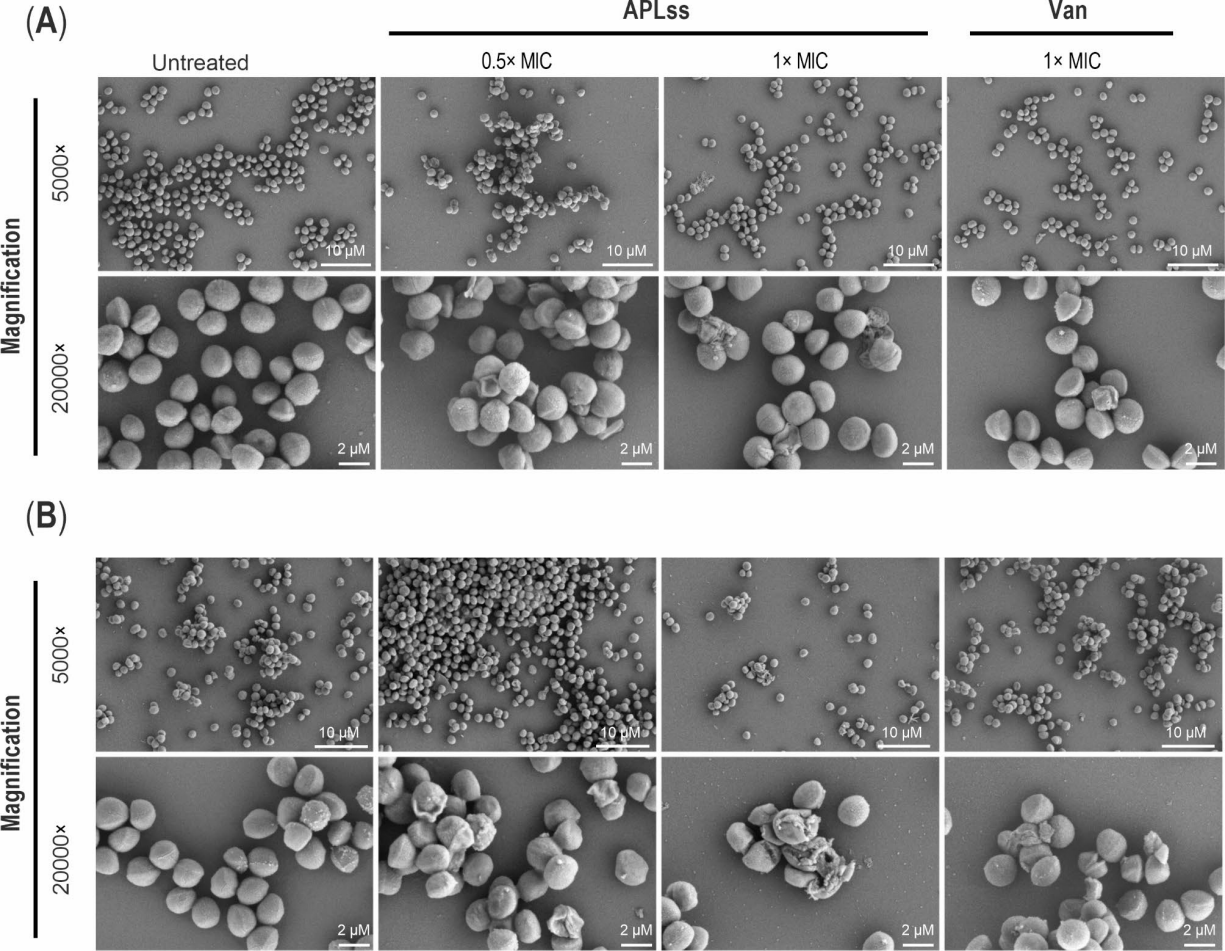
Scanning electron micrograph of APLss-treated staphylococcal strains **(A)** MSSA 25923 and **(B)** MRSA 33591. Staphylococcal cells were exposed to SEM following treatment with 0.5× and 1× MIC of APLss or 1× MIC of Van. In the left panel, untreated control cells have intact smooth surfaces. Scale bar: 2, 10 μm. Abbreviations: APLss, antimicrobial peptidase lysostaphin; MIC, minimum inhibitory concentration; Van, vancomycin

To further illustrate the bactericidal effect of APLss on the bacterial surface, we chose two reference strains, MSSA 25923 and MRSA 33591, based on their vegetative cell growth characteristics, and evaluated them using SEM. As shown in Fig. 3, while the bacterial cells in the control condition remained normal in size with smooth and intact surfaces, the APLss treatment caused a variety of damages in a dose-dependent manner, including cell shrinkage, fragmentation, lysis, and surface membrane rupture. Bactericidal activity, as represented by the time-kill curve, was observed to correlate favorably with APLss concentrations, whereas vancomycin at MIC demonstrated a lesser degree of destruction, with only modest shrinking or blisters on their surfaces. Altogether, the results from SEM suggested that there might be different mechanisms of cell inactivation from APLss and vancomycin treatment. Subsequently, the MICs of antimicrobial agents for each respective staphylococcal strain were utilized as a platform to assess their effect on growth and virulence factors.

### APLss inhibits staphylococcal adhesion to HVEC

**Fig. 4:**
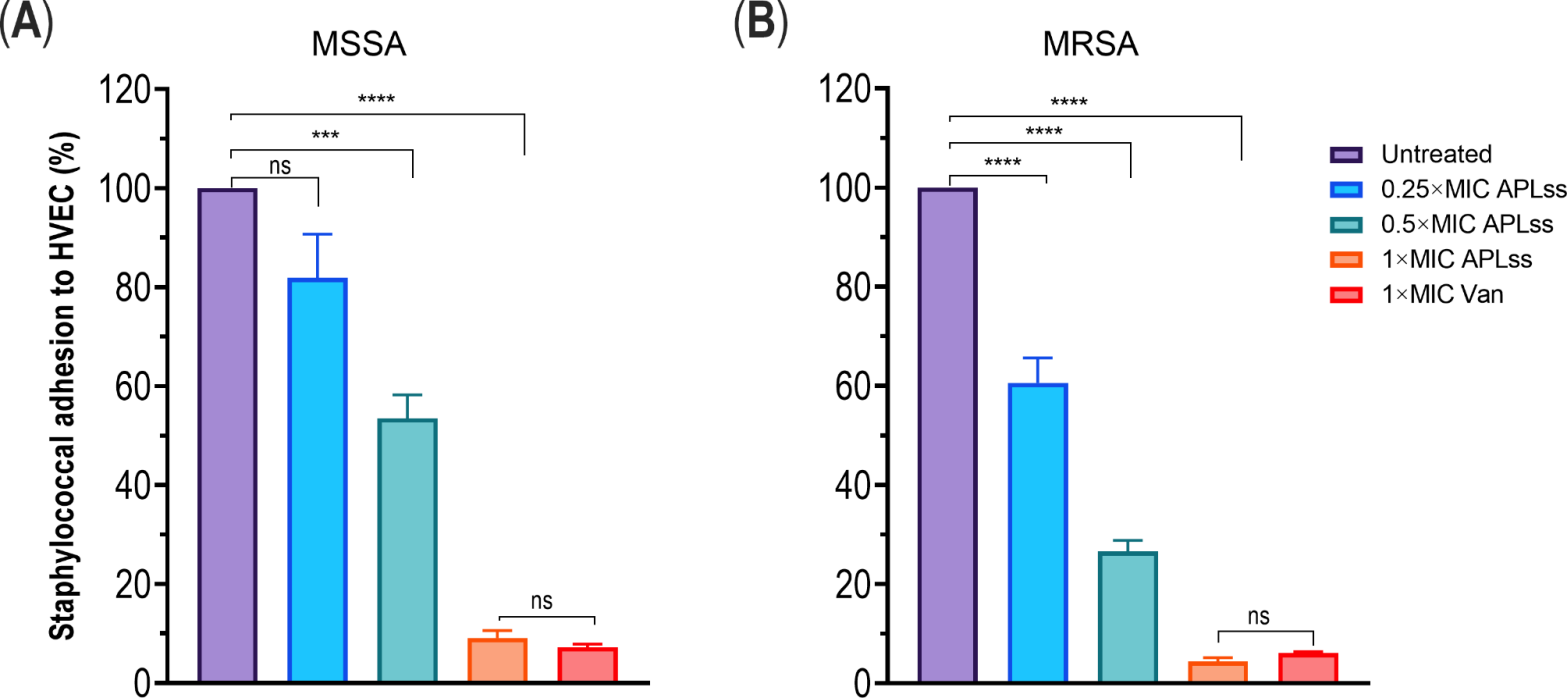
Adherence of staphylococcal strains **(A)** MSSA 25923 and **(B)** MRSA 33591 to HVEC. Subconfluent HVEC cell monolayers were infected with a suspension at a cell: bacterial ratio of 1: 200 in the presence or absence of varying concentrations of APLss or Van. HVEC were lysed to release any clinging bacteria, and then plated in serial dilutions on LB-agar plates. The bacteria adhering to the HVEC cells were quantified. The data are shown as mean ± SEM. Bars marked with a (*), (***) or (****) indicate statistical significance at the p < 0.05, p < 0.001, and p < 0.0001 levels, respectively. Abbreviations: APLss, antimicrobial peptidase lysostaphin; HVEC, human vaginal epithelial cells; MRSA, methicillin-resistant *Staphylococcus aureus*; MSSA, methicillin-sensitive *Staphylococcus aureus*; Van, vancomycin

Since staphylococcal strains are common colonizers of the vaginal epithelium and consequent causes of TSS, we investigated the potential of APLss to prevent staphylococcal strains’ adherence to lower female reproductive tract epithelial cells, HVEC. Two representative strains, MSSA 25923 and MRSA 33591 were employed in the quantitative adherence assay as described in the materials and methods. The antimicrobial agents tested, including APLss and Van at 1× MIC, were not cytotoxic to HVEC. Therefore, additional experiments were conducted to determine the effect of APLss on adherence, and the result showed that APLss dose-dependently suppressed staphylococcal adhesion to HVEC for both strains, with sub-MIC doses inhibiting adherence by 10–45% for MSSA 25923 (Fig. 4A) and 40–75% for MRSA 33591 (Fig. 4B). The effect of APLss was more prominent for MRSA 33591 at sub-inhibitory dose, indicating that APLss activity varies between strains. Notably, APLss was effective in preventing cell adherence at the MIC level, suggesting a potential role for APLss in in vivo application.

### APLss inhibits biofilm formation and facilitates established biofilm degradation

**Fig. 5:**
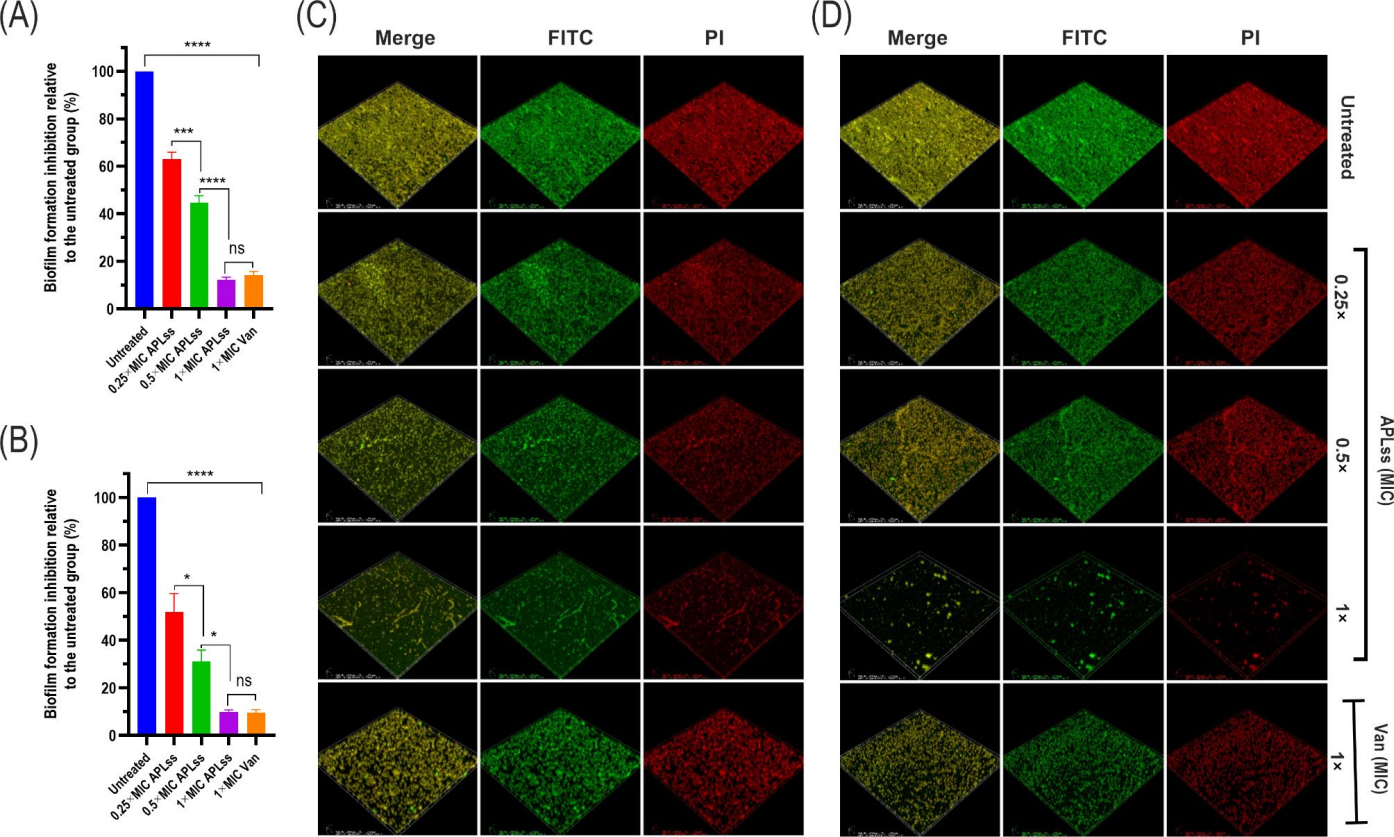
Biofilm biomass estimation using CV staining and fluorescent imaging. Staphylococcal strains were allowed to form biofilm in 96-well plates for 24 h in 200 µl of LB medium, which was supplemented with 0.25×, 0.5×, and 1× MIC of APLss or 1× MIC of Van. Percentage inhibition of biofilm grown in the presence of APLss or Van in comparison to the untreated control using CV staining is shown for strains including **(A)** MSSA 25923 and **(B)** MRSA 33591. Subsequently, fluorescent images were captured after staining with FITC and PI to visually assess the extent to which biofilm biomass was inhibited for included strains. FITC staining revealed green fluorescence from biofilm extracellular polysaccharides, and PI staining revealed red fluorescence from bacterial DNA. The data are presented as mean ± SEM. Differences between groups were evaluated using one-way ANOVA followed by Tukey’s multiple comparison test, and bars labeled with a (*) or (****) indicate statistical significance at the p < 0.05 and p < 0.0001 levels, respectively. Abbreviations: APLss, antimicrobial peptidase lysostaphin; FITC, fluorescein isothiocyanate; MIC, minimum inhibitory concentration; ns, not significant; PI, propidium iodide; Van, vancomycin

The ability of staphylococcal strains to adhere and form biofilm triggers an increase in bacterial virulence [34]. Hence, the efficacy of APLss on biofilm formation was further studied at both the sub-MIC and MIC levels. The biofilm biomass quantification assay demonstrated that APLss inhibited biofilm formation at 0.25× MIC and 0.5× MIC by 35% and 45%, respectively, for MSSA 25923 (Fig. 5A), and 55% and 75%, respectively, for MRSA 33591 (Fig. 5B). At the MIC level, the effect of APLss on biofilm inhibition was highest for both strains, although it was more pronounced for strain 33591, demonstrating that APLss activity differs between strains. Further, the biofilm formation inhibition effect of APLss was validated using FITC/PI staining and CLSM imaging (Fig. 5C and D). The extracellular polysaccharides of the biofilm fluoresced green with FITC staining, the intercellular spaces fluoresced red with PI staining, and the merge panel indicated the formation of extracellular FITC-reactive polysaccharides in intracellular gaps (Fig. 5C and D, merge panel). This demonstrated that extracellular polysaccharide was formed as a capsular component and appeared to cover the entire surface, implying that the artificial biofilms resembled the natural biofilms formed in lesions. Staphylococcal strains generated a thick biofilm composed of aggregates, with cells linked to one another in a 3-dimensional framework. In the absence of APLss, the biofilm was uniformly distributed and densely packed, covering the entire surface (Fig. 5C and D, untreated). However, following APLss treatment, the biofilm biomass of staphylococcal strains was noticeably diffuse with lower thickness and the cells scattered in treated samples, with APLss at 1× MIC having the maximum action for both representative strains. APLss inhibited biofilms of a representative MRSA strain more strongly than biofilms of a representative MSSA strain, supporting the results of the biofilm biomass assay. These findings demonstrated that APLss may significantly reduce staphylococcal biofilm formation during the initial adhesion stage in a dose-dependent fashion.

**Fig. 6:**
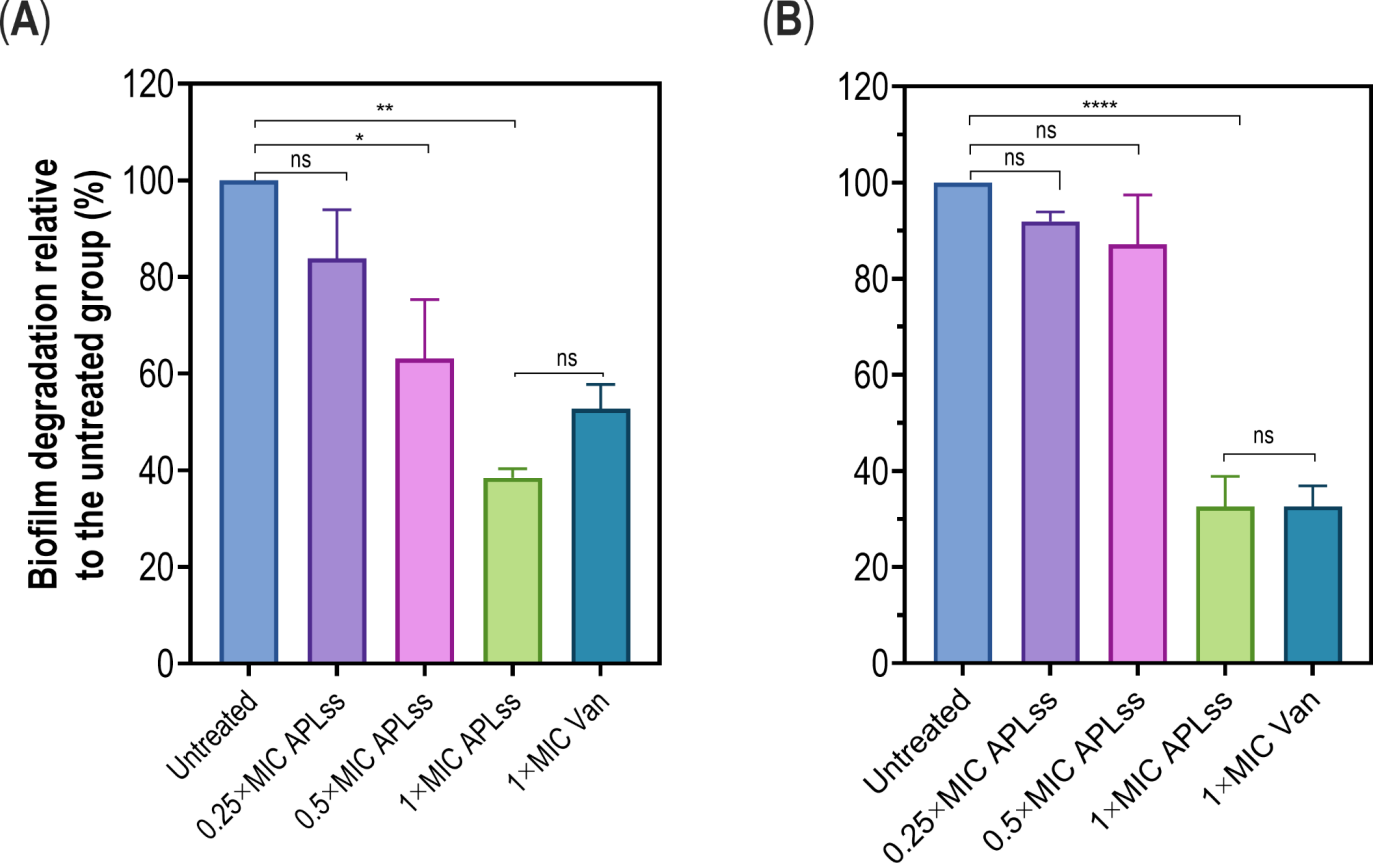
Quantification of biofilm biomass degradation using CV staining. Staphylococcal strains were allowed to form biofilm in 96-well plates for 24 h. The non-adherent staphylococcal cells were removed, and the established biofilm that adhered to the flat-bottomed 96-well plate was washed twice with PBS before being exposed for 3 h to 0.25×, 0.5×, and 1× MIC of APLss or 1× MIC of Van. The relative percentage of biofilm biomass in the presence of APLss or Van in comparison to the untreated control is shown for **(A)** MSSA 25923 and **(B)** MRSA 33591. The data are shown as mean ± SEM. Bars marked with a (*), (**) or (****) indicate statistical significance at the p < 0.05, p < 0.001, and p < 0.0001 levels, respectively. Abbreviations: APLss, antimicrobial peptidase lysostaphin; ns, not significant; Van, vancomycin

After establishing the effect of APLss against growing biofilm, we were then interested in studying its effect against mature biofilm. To this aim, preformed biofilms from strains 25923 and 33591 were exposed to increasing concentrations of APLss, and their relative biofilm inhibition was tracked by measuring OD_600_ against the untreated control. Staphylococcal biofilm was shown to be reduced to varying degrees across the two strains tested with 0.25× MIC and 0.5× MIC of APLss compared to the control group, with 1× MIC of APLss demonstrating the most substantial reduction. In a nutshell, the breakdown of established biofilm was directly proportional to the APLss concentration (Fig. 6). Albeit the CV staining assay demonstrated that APLss could inhibit the mature biofilm, it is important to note that APLss had a greater inhibitory influence in preventing biofilm formation at the same dose, suggesting that mature biofilm is more resistant to antimicrobials.

### APLss represses virulence gene expression

**Fig. 7:**
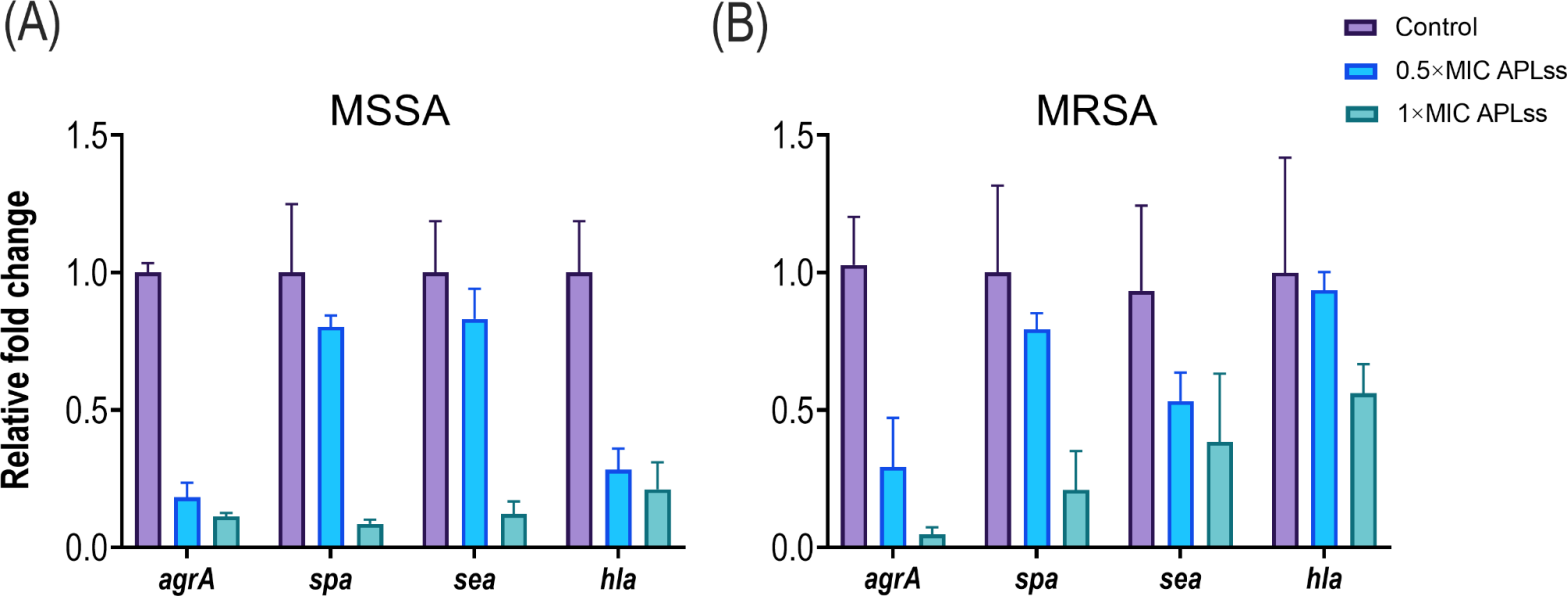
Transcriptional profiles of staphylococcal cells treated with or without APLss. Staphylococcal strains **(A)** MSSA 25923 and **(B)** MRSA 33591 were exposed to 0.5× and 1× MIC of APLss for 6 h. Transcriptional profiles were measured by qRT-PCR. Relative mRNA expression represents transcriptional fold changes of toxin genes vs. untreated controls. The experiment was performed in triplicate. Error bars represent standard errors of mean. The *, **, and *** denotes P < 0.05, P < 0.01, and P < 0.0001, respectively. Abbreviations: APLss, antimicrobial peptidase lysostaphin; MIC, minimum inhibitory concentration; MRSA, methicillin-resistant *Staphylococcus aureus*; MSSA, methicillin-sensitive *Staphylococcus aureus*

In an effort to gain insight into the molecular mechanisms underlying the antivirulence effects of APLss, we employed quantitative real-time polymerase chain reaction (qRT-PCR) to examine the differential expression of biofilm- and toxin-related genes in APLss-treated staphylococcal strains. The virulence characteristics of representative strains including MSSA 25923 and MRSA 33591 were investigated by using candidate genes like *agrA* (accessory gene regulator protein A), *hla* (α -hemolysin), *spa* (gene encoding staphylococcal protein A), and *sea* (staphylococcal enterotoxin type A). Table 1 lists the candidate gene primers, and the PCR-cycle parameters were as described in the methodology. At the MIC of APLss, gene expression levels for *agrA*, *hla*, *spa*, and *sea* in the MSSA strain were significantly downregulated (Fig. 7A). Albeit there was no statistically significant difference in *sea* and *hla* expression levels for the MRSA strain (Fig. 7B), the APLss effect in downregulating candidate gene expressions was apparent. It should be noted that the expression levels of *agrA* were significantly downregulated in both MSSA and MRSA strains, which is the parameter identified to connect with quorum sensing, and that quorum sensing positively influences biofilm formation and virulence attributes.

## Discussion

Staphylococci are commensal bacteria that reside on the epithelial cell surfaces of mammalian hosts and can cause serious illness when they break the epithelial barrier. The severity of the condition is exacerbated by the production of biofilms, which are complex structures that confer enhanced resistance to chemotherapeutics and host defenses, making illnesses hard to eliminate [4]. Recently, the dramatic rise in bacterial resistance to existing antibiotics has stimulated renewed efforts to the consideration of alternative treatment options that can effectively combat multidrug-resistant strains [35]. Bacteriocins are ribosomally-synthesized antimicrobial peptides/proteins that are naturally produced by various groups of bacteria to kill other bacteria. These proteinaceous toxins exhibit antimicrobial activity against several foodborne and human pathogens with various modes of action, including targeted nuclease activity, cell wall interference, and cytoplasmic membrane pore formation [35]. Due to their therapeutic properties, heat stability, low toxicity, and feasibility in production and modification, they are considered safe alternatives to traditional antibiotics. Furthermore, because bacteriocins, such as lysostaphin, are produced as gene-encoded proteins, they are infinitely more suited than classical antibiotics to bioengineering which could lead to the generation of a new source of potent antimicrobials [36]. Here, using MSSA and MRSA strains, we set out to investigate for the first time the ability of APLss at subinhibitory concentrations, to modulate both staphylococcal adherence to HVEC and virulence determinants, with the ultimate aim of targeting TSS-like life-threatening condition.

Initially, the ~ 28-kDa His-tagged target protein was overexpressed in *E. coli*, verified via Western blotting, and purified using Ni^2+^-NTA HisTrap column (Fig. 1). Our present strategy for soluble expression and IMAC purification of the His-tagged mature APLss was fairly effective in terms of recovery yield, purity, and ease when compared with other relevant publications that still have a constraint in one of these areas [37–39]. This purified protein was then utilized to determine the MICs of MSSA and MRSA strains. The results showed that APLss has a MICs range of 1.0–2.0 µg/mL against all tested bacteria (Table 2). While the MBCs of APLss for all tested isolates were between 2.0 and 4.0 µg/ml, which was less than 4 times the MICs of APLss, demonstrating the bacteriocin’s bactericidal action. In accordance with the MICs test, time-kill kinetics measured in reference staphylococcal strains showed a decrease in bacterial density after exposure to APLss. These findings are comparable with the previous study’s findings, which revealed that 2.0 µg/ml of lysostaphin inhibited *S. aureus* and MRSA within 2 h with a concurrent decrease in turbidity (OD_600_) of the cell suspensions [29]. It should be emphasized that the kinetic studies were followed for 2 h while the MIC determinations were performed for 24 h, so the difference in the susceptibility patterns could be demonstrated between each time interval rather than at one endpoint. Notably, exposure to APLss caused a time-dependent rise in the population of dead cells, providing more evidence for APLss’s activity as a bactericidal agent. We later hypothesized that APLss might operate on the bacterial membrane in a manner analogous to that of known membrane disruptor bacteriocins like nisin and polymyxin B, which have been shown to disperse membrane integrity [40]. In order to gain a better understanding of how they inhibit bacterial growth, SEM analysis was conducted, and the results suggested that the mechanism of cell inactivation from APLss and vancomycin treatment could be distinct. SEM micrographs revealed that lysostaphin potentially acts on the cell surfaces and causes significant membrane disruption on staphylococcal strains in a concentration-dependent fashion, but vancomycin had little or no effect, even at 1× MIC (Fig. 3). This finding was consistent with prior findings for Polygonum Chinese L. aqueous extract therapy of *S. aureus* [41]. It should be noted that the integrity of the bacterial cell surface is critical not only for cell protection but also for housing enzymes involved in cellular functions like the production of energy. Therefore, cell membrane breakdown could disturb cellular processes, affecting staphylococcal strain adhesion and growth.

Bacterial adhesion to host mucosal epithelial cells is a critical initial step in the pathogenesis of bacterial infection [42]. Antibiotic concentrations less than the MIC could indeed induce alterations in bacterial features such as physiologic and biochemical functionalities [21]. Therefore, we investigated the subinhibitory effect of APLss on staphylococcal adhesion to HVEC. The results demonstrated that staphylococcal strain adherence was dramatically decreased after APLss exposure (Fig. 4). Notably, even at concentrations lower than the inhibitory dose, APLss could effectively diminish staphylococcal strain adherence to the vaginal epithelium. The study of antimicrobial sub-MIC on adhesion is crucial because certain medications reach epithelial cell surfaces intermittently during therapy and may interfere with the pathogen’s ability to infiltrate tissue cells. Additionally, antimicrobials that impact the development or expression of bacterial molecules needed for adherence may substantially aid in unraveling the genetic and biochemical bases of bacterial contributing factors for host mucosa colonization. Traditionally, studies have focused on bacterial adhesion to fibronectin, [43] which could potentially obscure a range of additional adhesive processes [44]. Numerous studies have used microscopic or radioactively labeled bacterial techniques to estimate staphylococcal cell adhesion to epithelial cell surfaces [45]. To estimate staphylococcal adherence to human epithelial cells, scientists recently devised a high-throughput microtiter plate-based phenotypic assay that uses fluorescent tagging of the eukaryotic cell nucleus and microbes after adherence [45]. Nonetheless, despite screening thousands of chemicals, no effective in vivo candidate was found. We believe our approach is a pragmatic and effective way to uncover staphylococcal adhesion to host epithelium. The advantage of using in vitro testing to study bacteriocin’s impact is that the influence of antimicrobials on the adherence process can be easily monitored. Nevertheless, the majority of the physiologic restrictions of the intact endothelium surface are avoided. These issues are important to consider when extrapolating results from in vitro experiments to in vivo settings.

Staphylococcal adherence to eukaryotic cell surfaces is favorably associated with biofilm-forming capabilities, and strains having a higher propensity to form biofilm are more likely to be virulent [46]. Vancomycin, one of the most often used antimicrobials in the therapies for staphylococcal infection, was found to be ineffective against staphylococcal biofilms [47, 48]. Vancomycin apparently offers better therapeutic effectiveness against pre-formed staphylococcal biofilms when taken at a high dose and for an extended period of time [49]. Therefore, the ability of APLss to reduce biofilm formation and mature biofilm degradation was evaluated. The results showed that the sub-inhibitory concentration of APLss was highly effective in preventing biofilm formation (Fig. 5). We also discovered that the MRSA strain was more vulnerable to the influence of APLss than the MSSA strain, particularly in terms of biofilm formation, indicating that APLss activity varies between strains. Nonetheless, we genuinely believe that the virtually total reduction of biofilm formation at the MIC level of APLss is due to the prevention of bacterial cell growth rather than biofilm formation, given there were hardly any viable cells observed after the intervention. When compared to previous research on the effect of antimicrobial agent subinhibitory concentrations on biofilm formation, our findings contradicted the findings of Schilcher et al. [26], who discovered that subinhibitory doses of clindamycin actually promoted staphylococcal biofilm formation. A similar finding on the effect of sub-MIC doses of cloxacillin, cefazolin, and clindamycin on inducing biofilm formation in close-strain *S. epidermis* has been documented [50]. Furthermore, we investigated the effect of APLss on staphylococcal established biofilms; the findings revealed that APLss significantly disrupted staphylococcal established biofilms in a dose-dependent fashion when compared to the untreated control (Fig. 6). The current findings were compared to a previous study [51], which discovered that while alpha-mangostin at higher concentrations greatly inhibited *A. baumannii* initial stage biofilm formation, it was ineffective in inhibiting mature biofilms. Therefore, in an effort to gain insight into the molecular mechanisms, we examined the expression profiles of biofilm- and toxin-related genes in staphylococcal strains upon exposure to APLss.

Increasing evidence suggests that the sub-MICs of antimicrobial agents can alter the expression levels of bacterial toxins and those of factors responsible for other virulence attributes [52]. It has been established that exposure to β-lactams at sub-MICs increases the expression of staphylococcal exotoxins and adhesion elements [24]. Transcriptional analysis showed that the candidate genes such as *agrA*, *hla, spa*, and *sea* were downregulated upon APLss treatment in a concentration-dependent manner (Fig. 7). These findings are consistent with previous research in which a terpenoid (+)-nootkatone inhibited the expression of *sarA*, *agrA*, *RNAIII*, and *spa*, which regulate the expression of toxins, in both MRSA and MSSA strains [53]. Another study found that sub-MIC concentrations of clindamycin and linezolid consistently suppressed virulence genes like *hla* and *spa* expression [54], which was consistent with our findings. In TSS isolates, cytolysin *hla* is the major epithelial proinflammatory exotoxin that disrupts tissue and promotes biofilm formation, both of which influence staphylococcal phenotypic growth on vaginal mucosa [55]. Albeit there was no statistically significant difference in *sea* and *hla* expression levels for the MRSA strain (Fig. 7), the APLss effect in downregulating virulence gene expressions was apparent. Notably, *agrA* expression was found to be significantly downregulated in both MSSA and MRSA strains; this parameter was linked to quorum sensing, and quorum sensing has been shown to positively influence biofilm formation and virulence attributes like hla production [56–59]. Results from animal models and human cases of serious necrotizing illnesses caused by group A streptococcus and clostridial species have demonstrated that toxin suppression improves outcomes [60, 61]. Given the strong relationship between toxin production and serious infections in individuals, APLss could be useful in treating staphylococcal infections.

## Conclusion

The current findings show that APLss exhibits potent anti-staphylococcal activity by disrupting cell surfaces. Furthermore, even at subinhibitory levels, lysostaphin subjugated bacterial adhesion to the vaginal epithelium, biofilm formation, mature biofilm, and toxin production. Analysis of transcripts revealed that lysostaphin downregulates the expression of genes including *agrA*, *hla*, *spa*, and *sea*, thereby impeding biofilm formation and toxin production. Considering the potent inhibition properties of lysostaphin at multiple levels, our study provides new insight and evidence for bacteriocin research and the risk of sub-MIC to appropriate antibiotic administration by physicians and merits further investigation as a potential anti-staphylococcal agent with an antiadhesive mechanism of action using in vivo models of staphylococcal TSS.

### Electronic supplementary material

Below is the link to the electronic supplementary material.

Supplementary Material 1

Supplementary Material 2

Supplementary Material 3

## Data Availability

The manuscript includes all data generated during this study.

## Abbreviations

APLss: Antimicrobial peptidase lysostaphin
ATCC: American type culture collection
MBC: Minimum bactericidal concentration
MIC: Minimum inhibitory concentration
MRSA: Methicillin-resistant *Staphylococcus aureus*
MSSA: Methicillin-sensitive *Staphylococcus aureus*
