# Supplementary figures and images for: Antimicrobial peptidase lysostaphin at subinhibitory concentrations modulates staphylococcal adherence, biofilm formation, and toxin production
Source: BMC Microbiol. 2023 Oct 26;23:311. doi: 10.1186/s12866-023-03052-z (PMC10601153; doi:10.1186/s12866-023-03052-z)

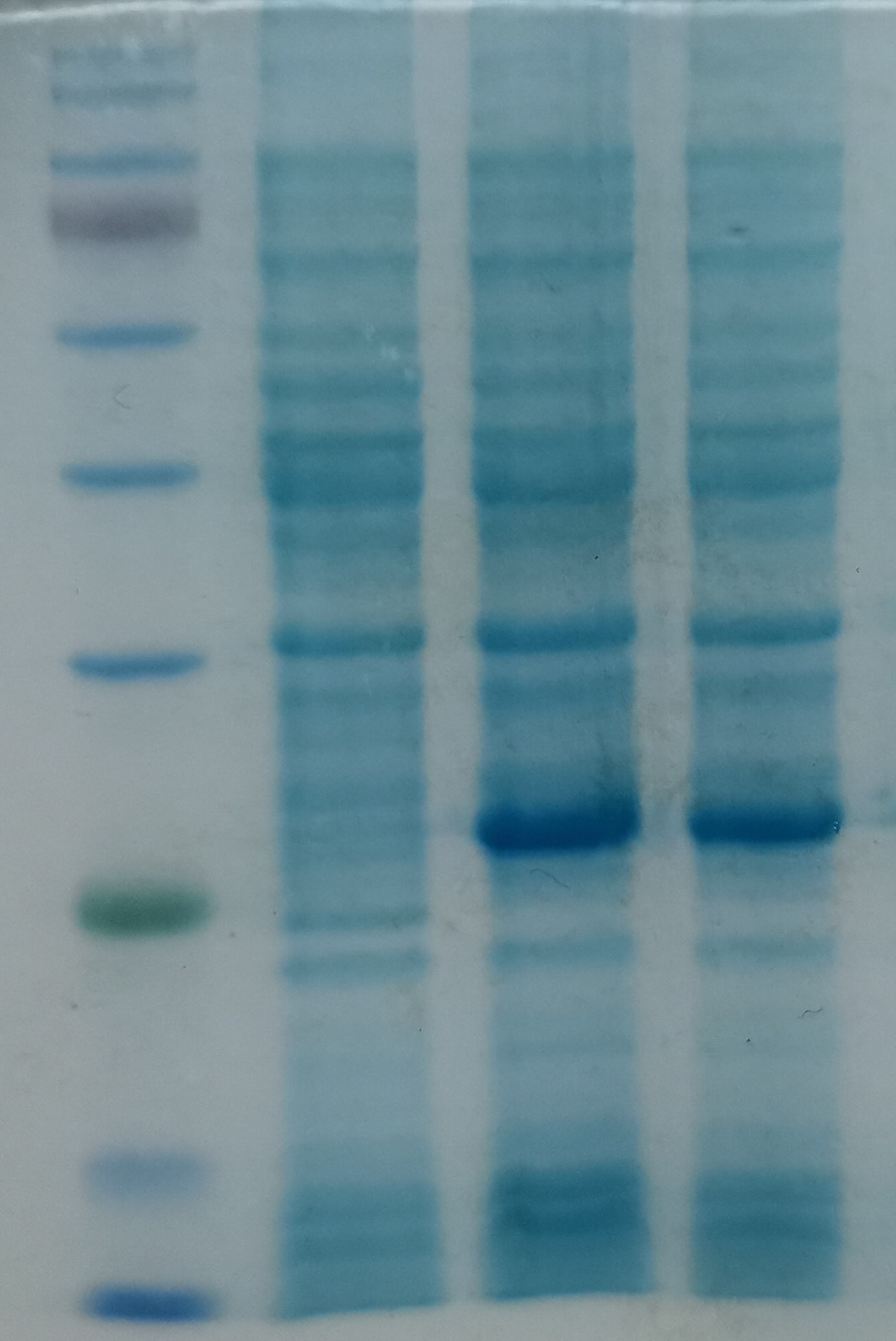

Supplement: Supplementary file 1 — Supplementary Material 1 [file 12866_2023_3052_MOESM1_ESM.jpg]

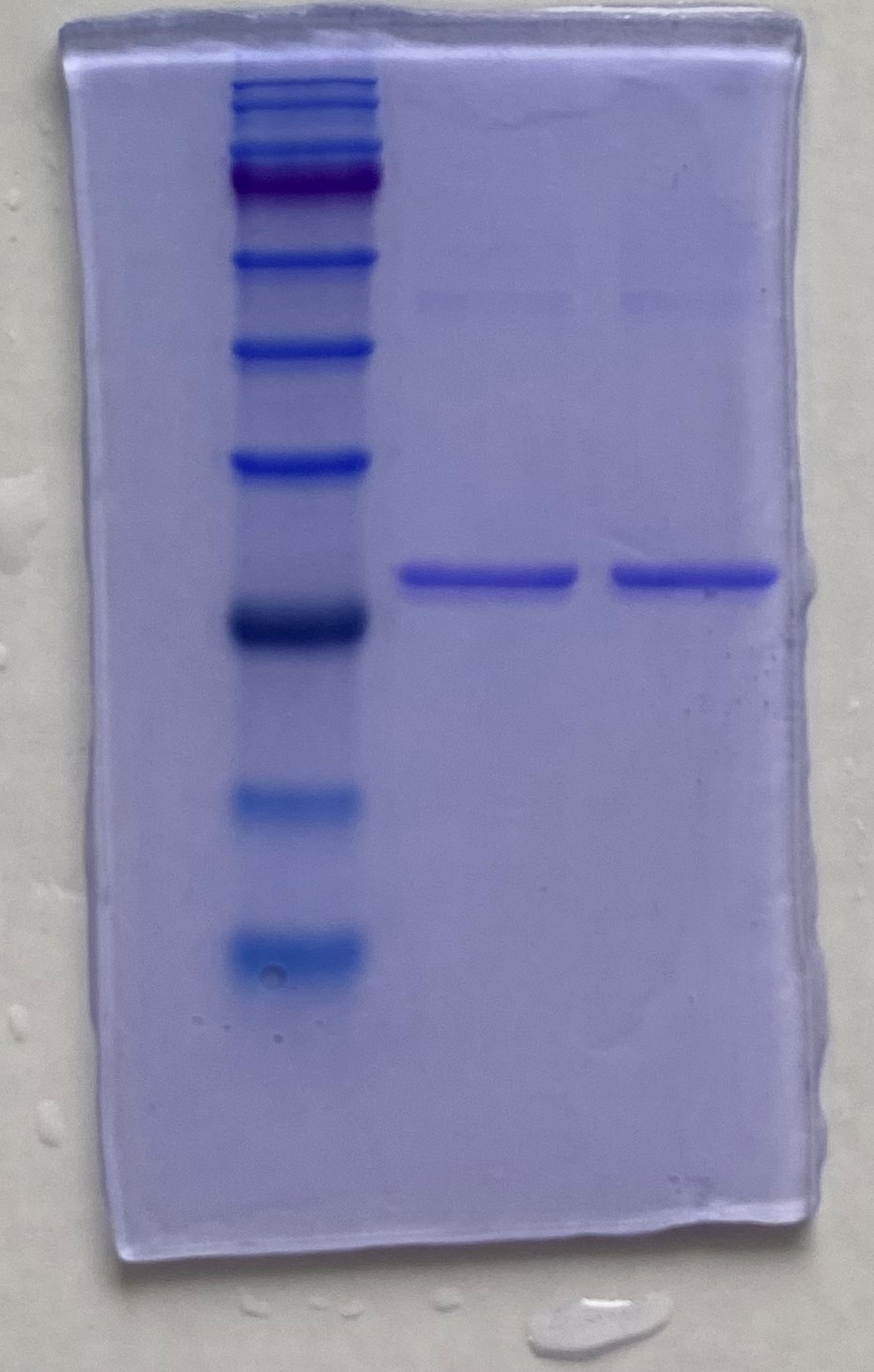

Supplement: Supplementary file 3 — Supplementary Material 3 [file 12866_2023_3052_MOESM3_ESM.jpg]
